# Impact of heat and high-moisture pH treatments on starch digestibility, phenolic composition, and cell bioactivity in sorghum (*Sorghum bicolor* L. Moench) flour

**DOI:** 10.3389/fnut.2024.1428542

**Published:** 2024-08-08

**Authors:** Jaymi Peterson, Adina L. Santana, Sarah Cox, Mayra Perez-Fajardo, Jose Covarrubias, Ramasamy Perumal, Scott Bean, Xiaorong Wu, Weiqun Wang, Dmitriy Smolensky

**Affiliations:** ^1^Grain Quality and Structure Research Unit, Agricultural Research Service, U.S. Department of Agriculture, Manhattan, KS, United States; ^2^Department of Grain Science and Industry, Kansas State University, Manhattan, KS, United States; ^3^Agricultural Research Center, Kansas State University, Hays, KS, United States; ^4^Department of Food Nutrition Dietetics and Health, Kansas State University, Manhattan, KS, United States

**Keywords:** sorghum flour, sorghum polyphenols, starch digestibility, sorghum bioactivity, high-moisture heat treatment, pH treatment

## Abstract

Sorghum (*Sorghum bicolor* L. Moench), characterized by substantial genetic diversity, encompasses some lines rich in health-promoting polyphenols. Laboratory studies have demonstrated anticancer properties of sorghum phenolics; however, their presence may impact nutritional factors, such as digestible starch. The objective of this study was to determine the effects of pH and high-moisture heating on starch digestibility, phenolic profile, and anticancer activity in sorghum. High Phenolic sorghum flour line SC84 was combined with buffer solutions (pH 3, 4, 5, 7, and 8) and heated for 0, 10, 30, 60, or 120 min. Starch digestibility was assessed using the K-DSTRS kit from Megazyme. Changes in phenolic composition were analyzed using total phenolic content (TPC) and condensed tannin content (CTC) assays coupled with reversed phase high performance liquid chromatography (RP-HPLC) analysis. Anticancer potential against human colorectal cancer cells (HCT116 and SW480) was determined though cell viability assay. Results indicated a significant increase in total starch digestibility of sample after heating. Heating samples for 10 min did not significantly reduce TPC of samples. However, CTC was significantly reduced with heating time, while pH exhibited no significant effect on CTC. The measured 3-deoxyanthocyanidins experienced a significant decrease (*p* < 0.0001), while certain flavonoids increased significantly (*p* < 0.05) after heating for 30 min or longer. Notably, the 10 min heating duration minimally affected anticancer activity, whereas longer heat times diminished extract efficacy against human colorectal cancer cells. Alkaline pH levels significantly decreased anticancer activity, regardless of heating time. Importantly, heating sorghum for 10 min improved starch digestibility with minimal compromise to potential health benefits. These findings suggest promising implications for the development of high-phenolic sorghum products, and provide valuable insights to guide forthcoming animal and clinical studies. The demonstrated impact of wet-heating on increased starch digestibility, coupled with the preservation of phenolic content and bioactivity, underscores the potential of incorporating high-phenolic sorghum lines in future functional food formulations.

## Graphical abstract

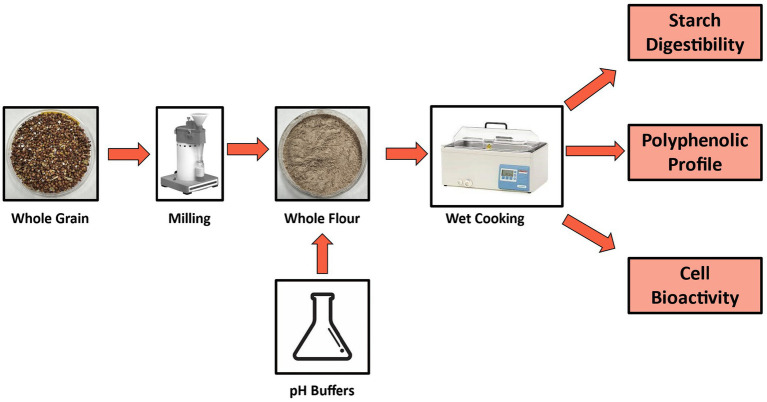

## 1 Introduction

Sorghum (*Sorghum bicolor* L. Moench) is a commonly grown dryland cereal crop originating from Africa (1). Sorghum is considered an agronomically advantageous crop for its drought tolerance and adaptability to grow in a varied of soil conditions and altitudes (2). According to a recent report from the Foreign Agricultural Service (FAS), the United States is the top producer of sorghum grain in the world. In 2023, the US produced 8,175 metric tons, ranking sorghum the 3rd most produced cereal grain in the country. In the US, Kansas and Texas produced 71% of the country’s entire sorghum production (3). Most of the sorghum produced in the US is used for animal livestock feed, exports to other countries, and ethanol production.

Sorghum is considered a food staple in many Asian and African countries, where it is used to make breads, porridges, and even fermented alcoholic beverages (4). Sorghum contains many major nutrients, including starch, protein, lipids, minerals, and vitamins. Additionally, sorghum is high in fiber, gluten free, and is a non-genetically modified organism (5). Despite its nutritional benefits, little sorghum grown in the US is used for human consumption.

Despite the underutilization of sorghum, the cereal grain has gained recent attention for containing health-promoting compounds called polyphenols. Polyphenols are secondary plant metabolites mostly found in fruits, vegetables, and grains. Polyphenols consist of multiple phenol units, and contain many different families of phenolic compounds defined by differences in their structure. Detected families of phenolic compounds reported in sorghum include phenolic acids, flavonoids, and condensed tannins. Polyphenols found in sorghum are reported to have many health benefits. One health benefit of importance is its anticancer property. For example, ethanolic sorghum bran extracts have shown antiproliferative properties in human adenocarcinoma and liver cancer cells (6), human colon cancer cells (7), and in colon cancer-induced mice models (8). Moreover, ethanolic extracts from whole sorghum flour have also shown effective cell growth inhibition in human hepatocarcinoma and colorectal cancer cells (9). In addition to the anticancer properties that have been demonstrated in sorghum derived polyphenols, they may also serve as natural alternatives for some synthetic compounds used in food manufacturing.

Other uses of sorghum derived polyphenols include serving as a natural source of antioxidants, food colorants, and dyes. For example, 3-deoxyanthocyanidins (3-DAs) have gained attention in the food industry as natural colorants and antioxidants, especially given their improved stability during processing, when compared to anthocyanins (10). They are water-soluble pigments responsible for either orange-red or blue-violet pigmentation in plants. During food processing, however, there are many factors that might affect the amount, structure, stability, and bioactivity of polyphenols found in sorghum. For example, defatting and deproteinating sorghum flour has been shown to reduce anti-oxidant activity (11).

If sorghum is to be fully utilized for its health benefits and agronomical advantages, it is imperative to understand the effects of processing on polyphenols and other nutritional components of high polyphenol-containing sorghum. Understanding the effect of processing on sorghum polyphenol composition may increase its potential for value-added or functional food formulations. The objective of this study was to determine the effects of heating time and pH on high polyphenol sorghum lines for starch digestibility, phenolic profile, and cell bioactivity in a high-moisture environment.

## 2 Materials and methods

### 2.1 Materials

Grain samples from high phenolic, high tannin brown sorghum grain (SC84) (12) were used in this study. Grain samples were grown in the winter nursery at Puerto Vallarta, Mexico, in 2018–2019 by the sorghum breeding program, Kansas State University, Agricultural Research Center (Hays, Kansas, United States). The reagents vanillin and Folin–Ciocalteu (FC) were purchased from Sigma-Aldrich (Milwaukee Wisconsin, United States). Absolute ethanol (grade USP), hydrochloric acid (HCl), Trolox, potassium phosphate monobasic and dibasic, gallic acid, and anhydrous sodium bicarbonate were purchased from Thermo Fischer Scientific (Waltham, Massachusetts, United States). ACS reagent grade absolute methanol was purchased from Ricca Chemical Company (Alrington, Texas, United States).

### 2.2 Sample preparation

Whole sorghum kernels were ground though a 0.5 mm screen using a UDY Mill (UDY Corporation Cyclone Sample Mill, Fort Collins, CO, United States). Sorghum flour was combined with 0.02 M sodium phosphate buffer solutions at pH 3, 4, 5, 7, and 8 at a ratio of 1:20 (w:v). Samples were placed in a water bath at 100°C and heated for 0, 10, 30, 60, or 120 min. Unheated samples (0 min) were used as a control. After the heating process, the entire samples (liquid and solid portions) were freeze-dried for 48 h and stored at −80°C until further use.

### 2.3 Starch digestibility

Digestible and resistant starch was measured using the K-DSTRS kit from Megazyme (Megazyme LTD, Ireland). Flour samples were incubated with continuous shaking in a sodium maleate buffer (50 mM, pH 6) with a mixture of pancreatic α-amylase and amyloglucosidase for 4 h at 37°C. Aliquots were removed at 240 min for digestible starch (DS) and resistant starch (RS). To measure DS, a 1 mL aliquot was added to 20 mL of 50 mM acetic acid in order to terminate the reaction. Then, 0.1 mL aliquots were incubated with 0.1 mL of amyloglucosidase (100 U/mL) at 50°C for 30 min. A final incubation with GOPOD reagent was carried out at 50°C for 20 min before absorbance was measured at 510 nm. To measure RS, a 4 mL aliquot was removed and added to 4 mL of ethanol (95% v/v). The samples were centrifuged at 4,000 rpm for 10 min. and the supernatant was discarded. The pellet was washed twice with aqueous ethanol (50% v/v) and then suspended in 2 mL sodium hydroxide (1.7 M) to dissolve the resistant starch. Then, 8 mL of sodium acetate buffer (1 M) was added to neutralize the solution. A portion (0.1 mL) of amyloglucosidase (3,300 U/mL) was added before incubating for 30 min at 50°C. Aliquots (0.1 mL) were incubated with GOPOD reagent at 50°C for 20 min before absorbance was read at 510 nm. Total starch (TS) content was calculated according to Equation 1 and reported in “as is” basis.


(1) DigestibleStarch+ResistantStarch=TotalStarchContent

### 2.4 Polyphenolic profile

#### 2.4.1 Total phenolic content

One gram of flour samples was combined with 9 mL ethanol (70% v/v) and placed on a shaker for 2 h. After stirring, samples sat overnight (18 h) at −20°C and were centrifuged for 10 min at 4°C at 2,970×*g* the next day. Supernatants were collected and analyzed for total phenolic content (TPC) using the Folin–Ciocalteu (FC) method as described by Herald et al. (13). Briefly, a standard curve of gallic acid ranging from 0 to 800 μg/L was used to quantify gallic acid equivalents (GAE) in mg per milliliter of sample. FC reagent was diluted in a 1:1 working ratio with DI water. Samples were diluted with 70% ethanol solvent to achieve acceptable absorbance values. The diluted sample (25 uL) was combined with 75 μL of Deionized (DI) water and 25 μL of FC reagent in a 96 well plate. After a brief 6 min incubation period, 100 μL of 7.5% (w/v) Na_2_CO_3_ was added to the each well simultaneously using a 96 channel semi-automated pipettor (Sorenson Bioscience Inc., Radnor PA, United States). Samples were sealed with heat seal film and incubated in the dark for 90 min. After the final incubation period, sample absorbance values were measured at 765 nm using a Biotkek synergy 2 multi-detection plate reader (Winooski, VT, United States). TPC was calculated based on the gallic acid standard curve and expressed as mg gallic acid equivalents (GAE) per g sample.

#### 2.4.2 Condensed tannin content

The condensed tannin content (CTC) of the samples were determined with slight modification using a previously published method (14). Briefly, 0.1 g of sample was extracted for 20 min in 1 mL 1%HCl-methanol at 20°C. Samples were centrifuged at 805 × *g* for 6 min and decanted. Aliquots were used for analysis and diluted (if needed) in 1%HCl-Methanol. Catechin standard was prepared by dissolving catechin in 100% (v/v) methanol at a concentration of 10 mg/mL. The calibration curve was diluted in range of 0–5.0 mg/mL catechin in 1%HCl-methanol. Both standards and samples were added (30 μL) to their corresponding wells in a 96-well plate. Vanillin reagent (1% vanillin w/v and 8% HCl v/v) (150 μL) and 4% HCl-methanol (150 μL) were added to the well plate using a 96 channel semi-automated pipettor (Sorenson Bioscience Inc., Radnor PA, United States). After incubating for 20 min at 30°C, absorbance values were measured at 500 nm using a Biotek synergy 2 multi-detection plate reader (Winooski VT, United States). The absorbance of the blank (no vanillin reagent) was subtracted from the corresponding standard or sample with vanillin and plotted against the standard curve to calculate catechin equivalents (CE) in mg per g flour.

#### 2.4.3 HPLC analysis

Phenolic profiles of samples were determined using HPLC methods previously described by Lee et al. (15) adapted from Irakli et al. (16) using a 1290 Infinity II (Agilent, Santa Clara, United States) HPLC coupled with a diode array detector.

A C18 column (Kinetex^®^, 2.6 μm, 100 Å, 150 mm × 4.6 mm size, Phenomenex, Torrance, CA, United States) attached to a guard column (SecurityGuard™, Phenomenex, Torrance, CA, United States) was used for sample analysis. The mobile phases contained acetonitrile (A), methanol (B), and water-glacial acetic acid, 0.5% v/v (C). The elution gradient flowed at 1 mL/min at a temperature of 30°C and consisted of: initial composition of 5:5:90 (A:B:C), followed by 0–5 min of 8:8:84, 5–15 min of 10:10:80, 15–25 min of 25:0:75, 25–35 min of 30:0:70, 35–45 min of 60:0:40. To stabilize the base line for future runs, a post-time of 5 min with the initial gradient was used.

Selected samples heated for 0, 10, 30, and 60 min at pH 3, 5, and 8 were analyzed in triplicate. Samples were combined with ethanol (70% v/v) at a ratio of 1:10 (w:v) and centrifuged for 10 min at 4°C at 2,970×*g*. Supernatants were collected and dried at 60°C (Rocket Synergy 2 Evaporation System, Thermo Fisher, Pittsburgh, United States). The dried extracts were mixed with 10 mL methanol: ethanol (90,10, v/v) in an ultrasonic bath (ULTRAsonik, Simi Valley, CA, United States) for 40 min. Samples were then passed through a 0.45 μm polyvinylidene difluoride (PVDF) syringe filter before injection.

Working solutions of phenolic standards were prepared in methanol: ethanol (90,10, v/v) for the calibration curves (9.00 × 10^−5^–0.09 mg/mL, R^2^ ≥ 0.99). The chromatograms at 280, 320, and 510 nm were selected. The standards eriodyctiol, protocatechuic acid, catechin, epicatechin, quercetin, luteolin, and naringenin were quantified at 280 nm. Apigenin, chlorogenic-, caffeic-, p-coumaric- and ferulic acids were quantified at 320 nm. Luteolinidin, apigenidin, and the 7-methoxyapigenidin were quantified at 510 nm. Target compounds were calculated based on standard curves and expressed as μg of target compound per g sample.

### 2.5 Cell bioactivity

To determine the cell bioactivity of sorghum phenolics, a cancer cell viability assay (MTS) was performed. Human colorectal carcinoma cells (HCT 116) and SW480 cells were grown in McCoys media and RPMI 1640 media, respectively, containing 10% v/v fetal bovine serum (FBS) and 1X antibiotic antimycotic were added to both media. Cells were maintained at 37°C and 5% CO_2_.

HCT116 and SW480 Cells were plated at 5,000 and 10,000 cells/well, respectively, in 96 well plate, and allowed to grow overnight before treatment. Cells were treated with 3 different concentrations of aqueous ethanolic extracts (20, 10, 5 mg/mL) with 70% ethanol serving as vehicle control. Cells were incubated for 48 h, and cell viability was measured using the MTS assay kit (Promega Cell Titer 96^tm^ AQ_ueous_ One Solution Cell Proliferation Assay) from Fisher scientific (Pittsburg, PA, United States). In short, the media was removed from the wells and the plates were washed with PBS. Media (100 μL) containing MTS reagent at 20 μL/mL was added to each well and incubated at 37°C and 5% CO_2_ for 1 h (HCT116) and 1.5 h (SW480). The plates were then read at 490 nm on a Biotek H4 Plate Reader (Winooski, VT, United States). Results are expressed as percent cell inhibition and were calculated according to Equations 2, 3.


(2) $$\begin{array}{l} \text{Cell}\;\text{viability}\;\left(CV\right)=\left(\text{Abs}\;\text{sample}-\text{Abs}\;\text{blank}\right)/\\ \;\left(\begin{array}{l} \text{Abs}\;\text{vehicle}\;\text{control}-\\ \text{Abs}\;\text{blank} \end{array}\right) \end{array}$$

(3) $$\%\text{Cell}\;\text{Inhibition}=\left(1-CV\right)\times 100$$

### 2.6 Statistical analysis

Data represents the averages of at least three independent experiments. Results were analyzed using 2-way analysis of variance (ANOVA) with Tukey’s *post hoc* test using GraphPad prism version 9.4.0 (GraphPad, San Diego, CA). Mean values and standard deviations were reported with level of significance at *p*-value less than 0.05. Statistical significance is represented by: *p ≤ 0.5, **p ≤ 0.1, ***p ≤ 0.01, and ****p ≤ 0.0.001. Pearson’s correlation coefficient, or Pearson’s r, was performed to examine the relationship of TPC and CTC on %Cell inhibition.

## 3 Results

### 3.1 Starch digestibility

**Figure 1:**
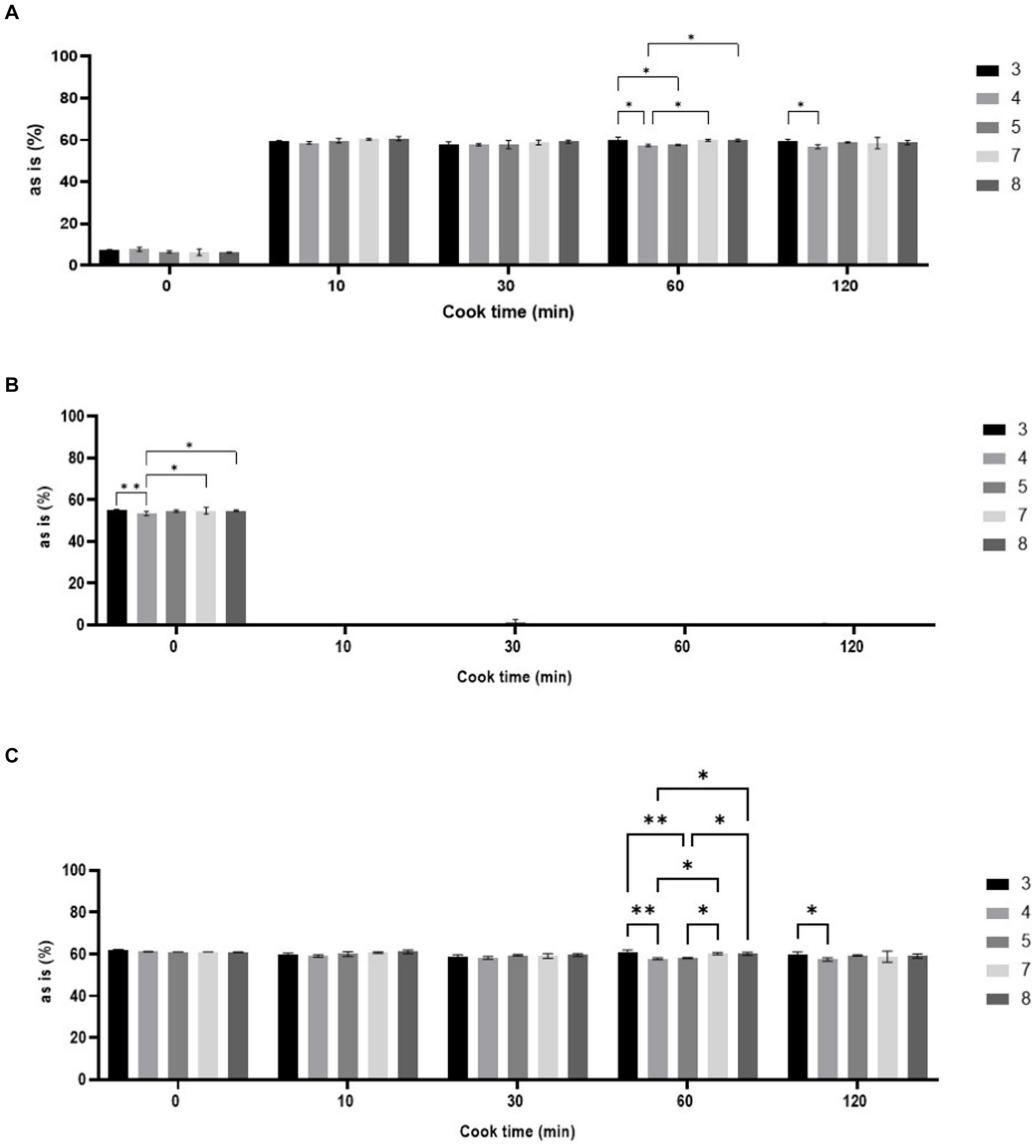
Overall changes in starch. Digestible starch **(A)**, resistant starch **(B)**, and total starch **(C)** in sorghum flour calculated on a % as is basis (g/100 g sample) after heat-moisture and pH treatments.

The digestible starch significantly increased (*p* < 0.05) from approximately 7 g/100 g flour in unheated samples to 60 g/100 g flour after 10 min of heating for all pH treatments. Heating the samples for longer than 10 min did not significantly affect total starch digestibility, regardless of pH treatments. The total starch digestibility was only affected by pH after heating for 30 min (Figure 1A). Resistant starch significantly decreased after heating for 10 min. Heating for longer than 10 min did not significantly affect resistant starch. Smaller, significant changes in non-digestible starch from the pH treatments were only detected in unheated samples (Figure 1B). The total starch content, which was calculated from the digestible and non-digestible starch fractions, was not significantly affected by heating, regardless of time. There were small, but significant, differences in total starch across pH groups for the 60 and 120 min time points (Figure 1C).

### 3.2 Polyphenolic profile

#### 3.2.1 Total phenolic content

**Figure 2:**
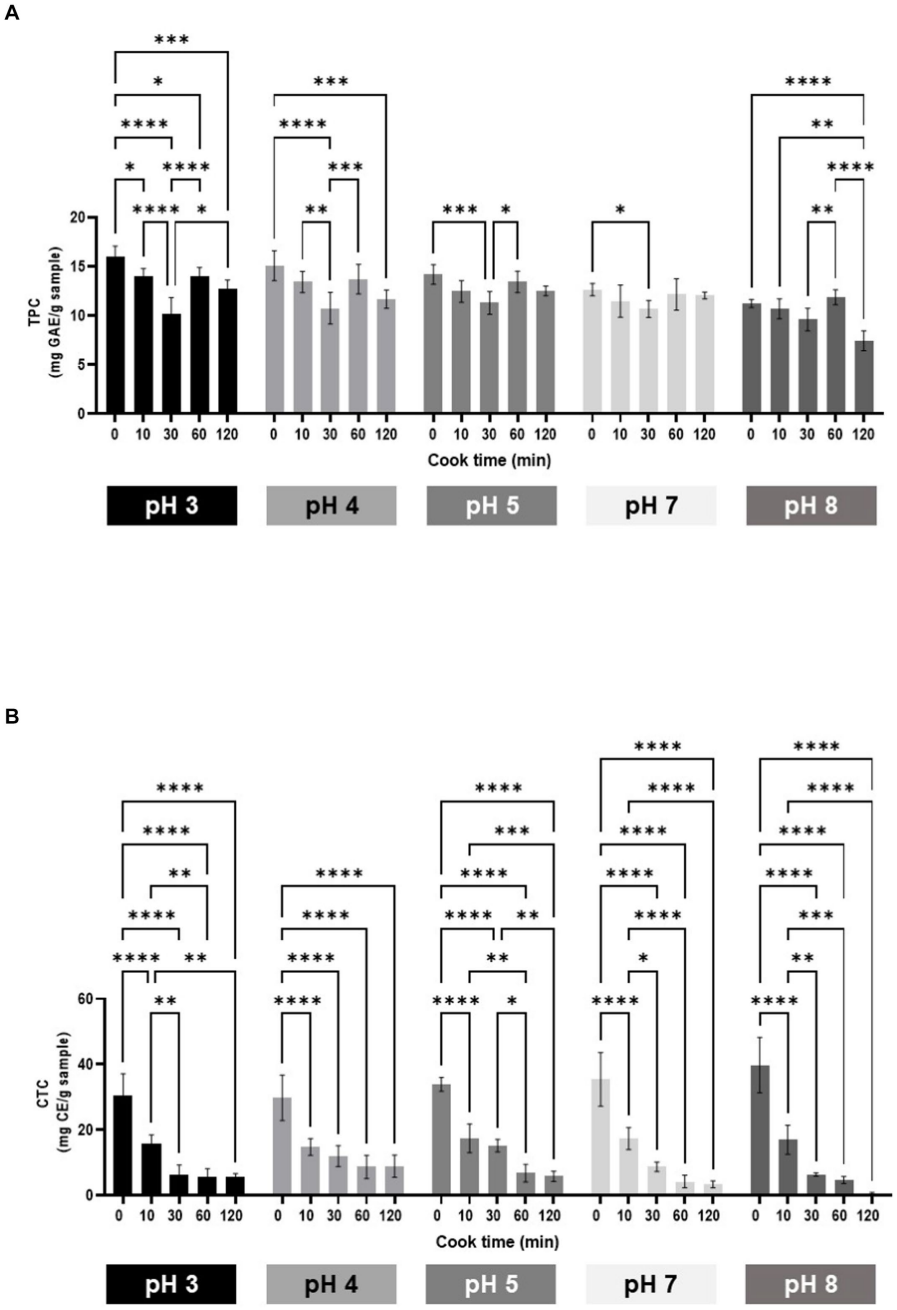
Phenolic profile. Total phenolic content **(A)** and condensed tannin content **(B)** of sorghum. Total phenolic content expressed as milligram gallic acid equivalents per gram (mg GAE/g). Condensed tannins content expressed as mg catechin equivalents per gram (mg CE/g).

The TPC of unheated samples ranged from 11.21 to 16.0 mg GAE/g across pH treatments. Heating the samples for 10 min did not significantly decrease TPC, with the exception of pH 3. TPC was significantly decreased after heating samples for 30 min, with exception of pH 8. Surprisingly, after heating for 60 min, there was a significant increase in TPC when, compared to 30 min for samples buffered at pH 3, 4, 5, and 8. Throughout the 120 min heating time, total phenolic content was most stable at pH 5 and 7 (Figure 2A). The overall TPC of samples at neutral and alkaline pH levels (7 and 8) were lower with exception of the 30 min heating group (Supplementary Table S1).

#### 3.2.2 Condensed tannin content

The CTC of unheated samples ranged from 29.77 to 39.76 CE (mg/g) across all pH treatments. Heating the samples for 10 min significantly decreased CTC across all pH groups by an average of 51.46% Heating the samples for longer than 10 min continued to decrease CTC across all pH groups (Figure 2B). Unheated samples buffered at pH 8 were significantly higher in CTC than samples at pH 3 and 4. Although unheated samples buffered at pH 8 started with a higher CTC as compared to pH 3 and 4, they were the lowest in CTC (0.46 mg CE/g) after heating for 120 min (Supplementary Table S1).

#### 3.2.3 HPLC analysis

Selected samples heated at pH 3, 5, and 8 were selected for further in-depth analysis using HPLC. The following phenolic compounds were detected: caffeic acid, ferulic acid, p-coumaric acid, luteolin, apigenin, catechin-, epicatechin, naringenin, eriodyctiol, taxifolin, quercetin, luteolinidin, apigenidin, and 7-methoxyapigdenidin.

**Figure 3:**
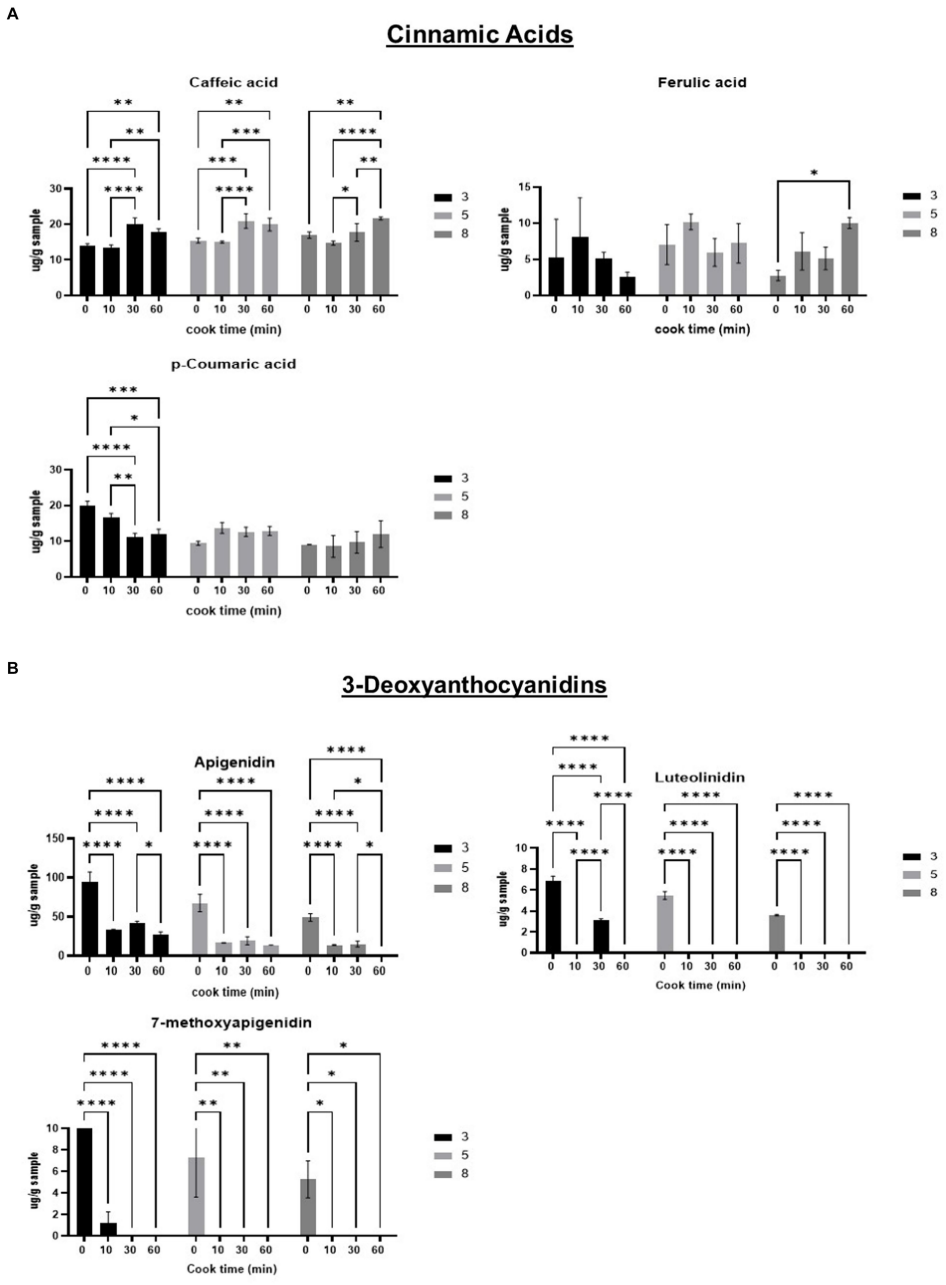

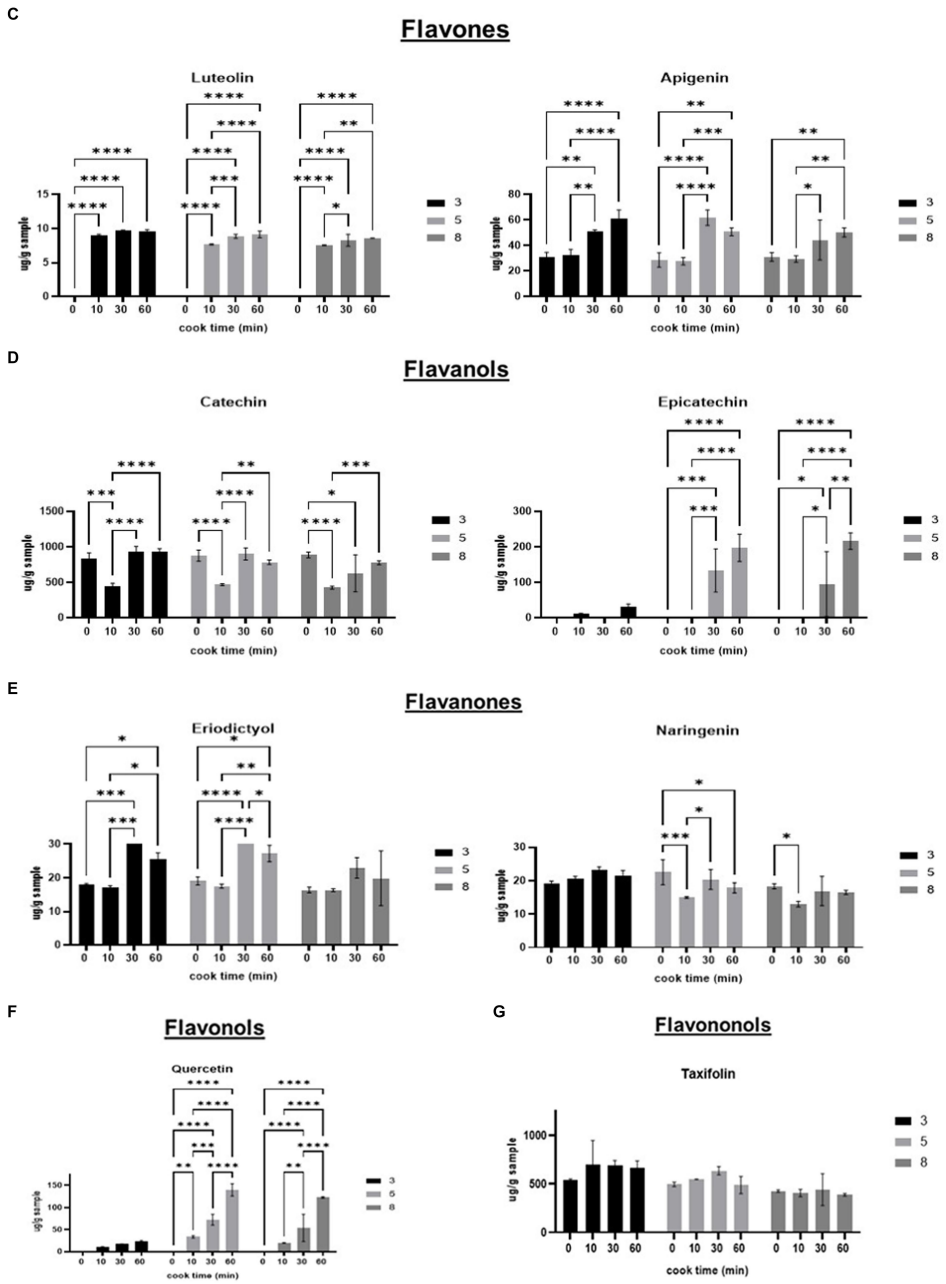
Phenolic composition of phenolic compounds detected in sorghum; Cinnamic acids **(A)**; 3-Deoxyanthocyanidins **(B)**; Flavones **(C)**; Flavanols **(D)**; Falvanones **(E)**; Flavonols **(F)**; Flavononols **(G)**. Phenolic compounds expressed as micrograms per g sample (ug/g).

Caffeic acid concentrations significantly increased after 30 min of heating for all pH groups. Ferulic acid significantly increased at 60 min, when compared to unheated control at pH 8. The compound p-coumaric acid significantly decreased over time in pH 3. The results indicated that cinnamic acids are more stable and/or increased by heating at higher pH (5 and 8), except for caffeic acid, which increased at all three pH levels (Figure 3A).

The detectable 3-deoxyanthocyanidins (3-DAs), apigenidin, luteolinidin, and 7-methoxyapigenidin all decreased after 10 min of heating, regardless of pH level. Heating for longer than 10 min continued to significantly decrease 3-DA content. Apigenidin was the most abundant 3-DA, with concentrations around 10 times higher than the other 3-DAs detected. Luteolinidin was detected in such small quantities that the signal to noise ratio is likely too high for results to be evaluated accurately (Figure 3B).

Both flavone compounds, luteolin and apigenin, increased after heating, regardless of pH. Luteolin was not detected in unheated samples and was detectable after 10 min of heating. Apigenin content significantly increased after 30 min of heating across all three pH groups (Figure 3C).

The flavanol compound, catechin, significantly decreased after 10 min of heating, but increased to unheated levels by 60 min. Epicatechin was not detectable in unheated samples, but increased significantly with heating time in samples buffered at pH 5 and pH 8, but not at pH 3, where the increase was not significant (Figure 3D).

The flavanone, naringenin, significantly decreased in concentration after 10 min of heating in samples buffered at pH 5 and pH 8 (Figure 3E). After 30 min, eriodyctiol, also a flavanone, significantly increased in concentration from 17.96 μg/g in unheated samples to 30.24 μg/g at pH 3, and from 19.05 μg/g to 35.53 μg/g at pH 5.

Quercetin content increased over heating time at pH 5 and 8, while at pH 3 the increase was not significant. Heating for 60 min significantly raised quercetin content from 0 μg/g in unheated samples to 139.95 μg/g, and 122.73 μg/g in samples buffered at pH 5 and pH 8, respectively (Figure 3F). Quercetin was the only detected flavonol.

The only detectable flavanonol was taxifolin. Taxifolin content did not significantly change with heat time, regardless of pH level (Figure 3G). Taxifolin content was significantly higher in samples buffered at pH 3, when compared to samples at pH 8 across all heat times (Supplementary Table S2).

### 3.3 Cell bioactivity

**Figure 4:**
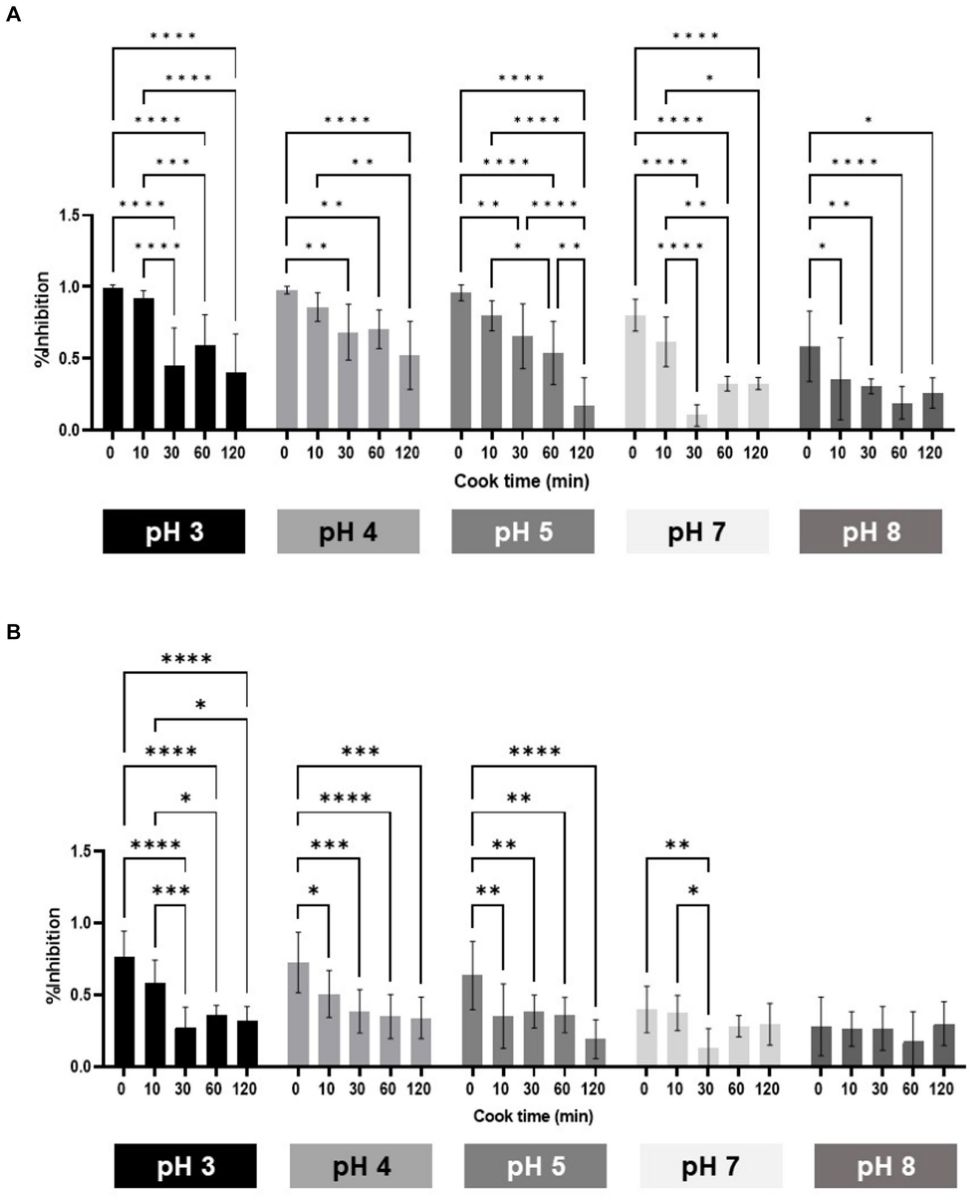
Cell bioactivity of phenolics on cell inhibition in HCT116 **(A)** and SW480 **(B)** human colon cancer cells.

Cell inhibition properties of extracts did not significantly decrease after heating for 10 min, with exception of pH 8 in HCT116 cells. Heating for 30 min significantly lowered cell inhibition of all samples regardless of pH level (Figures 4A,B). Samples buffered at pH 3, 4, and 5 were significantly higher in cell inhibition than pH 8 across all heat times, except for 120 min (Supplementary Table S3). Samples buffered at pH 8 had a significantly lower cell inhibition, when compared to pH 3 and pH 4 before and after heating samples for 10 min. After heating for 60 min, pH had no effect on cell inhibition of samples (Supplementary Table S3).

**Figure 5:**
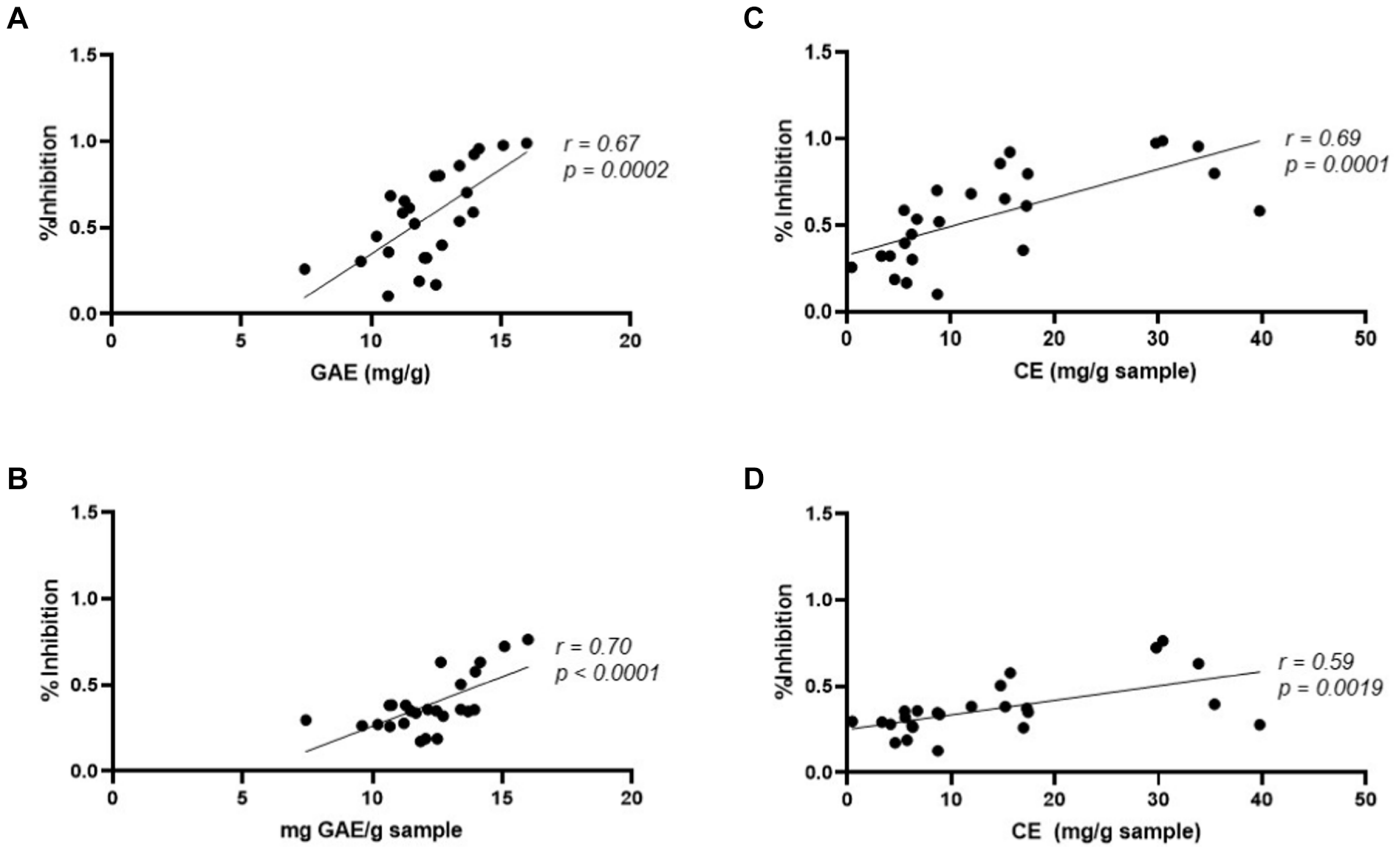
Pearson’s correlation of total phenolic content of **(A)** HCT116 and **(B)** SW480 and the condensed tannin content of **(C)** HCT116 and **(D)** SW480 cells compared to percent growth inhibition.

A correlation analysis to test whether total phenolic content and/or condensed tannin content was correlated to cell inhibition for both cell lines was carried out. Both analyses were overall positively correlated with percent cell inhibition for both cell lines. For HCT116, correlation for TPC was *r* = 0.67, while CTC was *r* = 0.69. For SW480 cells, TPC was *r* = 0.70 and CTC was *r* = 0.59 (Figure 5). Based on these results, both condensed tannin and polyphenols are correlated with cancer cell inhibition.

## 4 Discussion

### 4.1 Starch digestibility

In the present study, digestible starch significantly increased after heating, when compared to the non-heated samples in an excess moisture environment. The digestible starch of samples was around 60 g/100 g flour after 10 min of heating. This was comparable to Ezeogu et al. (17), who reported 85% digestible starch in a decorticated red sorghum variety after heating for 10 min. Differences in starch digestibility could be due to the presence (or lack of) specific polyphenolic compounds. Specific types of polyphenols impact starch digestibility differently depending on the type (ex; proanthocyanidins vs. monomeric phenolics) and polyphenolic concentration (18). The increase in digestible starch is inversely related to the resistant starch content in sorghum after heat and high-moisture pH treatments.

The amount of resistant starch in raw samples was around 55 g/100 g. The heat and high-moisture pH treatments presented in our study significantly reduced the resistant starch of our samples from 55 g/100 g to less than 1 g/100 g for all samples. This trend was also observed by Teixeira Nde et al. (19), where in RS of samples after wet-heating decreased by 94%. The same decrease in RS from heat and high moisture treatments were not reported by Vu et al. (20), who examined heat and moisture treatments in white sorghum flours and reported a 53–140% increase in resistant starch content after heat-moisture treatments. Differences in final resistant starch levels can be attributed to differences in methodology. Vu et al. evaluated high heat treatments with low-moisture levels for longer time periods, when compared to the current study. Other differences may be accounted for by differences in starting material composition and water content (excess of water vs. low moisture). For example, Vu et al. (20), who examined decorticated white sorghum flour commercially purchased from Archer Daniels, while the sorghum studied in our present study was high-polyphenol whole grain sorghum. The presence of pericarp present in whole grain flours will influence resistant starch content. In addition, pigmented sorghum grains are reported to have a higher initial RS content than white lines (21). Another factor that influences RS in grains is the proportion of water to grain during heat treatment. The greater the proportion of water present during the heating process, the greater degree of gelatinization, thus increasing digestibility of starch (19). To our knowledge, the direct effect of high-moisture pH heat treatments on starch digestibility has not been extensively studied in sorghum.

Total starch content for sorghum was around 60 g/100 g, which is comparable to the 70–75 g/100 g reported in Italian pigmented sorghum grain by Rocchetti et al. (21). Total starch content for sorghum may vary depending on genotype and cultivation conditions.

### 4.2 Polyphenolic profile

#### 4.2.1 Total phenolic content

The TPC of samples decreased, but not significantly after 10 min of heating for pH 4, 5, 7, and 8. It is commonly reported that wet-heating decreases TPC in sorghum samples (22). An additional study examining the effect of wet-heating (boiling) on sorghum phenolic content also reported a reduction in TPC by 18% in an unreported sorghum variety after 10 min of heating (23). A similar study performed by Sorour et al. (24) reported a 32 and 41% loss in TPC after boiling for 2 h in low-tannin and high-tannin sorghum lines, respectively. Differences in TPC reduction could be due to differences in variety, water ratio, and presence of buffer. For example, both studies by Li et al. (22) and Sorour et al. (24) mixed sorghum flour or grain with water at ratio of 1:5 (w/v). The methods described in our study examined wet-heating of sorghum flour to pH buffers at 1:20 (w/v) ratio and was performed with different sorghum lines than the previously reported. After heating for 30 min, samples decreased by 24.0% on average. TPC continued to decrease with longer heat times. Samples buffered at pH 8 were significantly lower in TPC than samples at pH 3, except for samples in the 30 min heating group. This is in line with the findings from Jurgens (25), who reported a 67% reduction in TPC from high pH levels in plant derived phenolics.

#### 4.2.2 Condensed tannin content

Unlike the TPC results, the CTC significantly decreased after 10 min of heating, regardless of pH level. Overall, the CTC reduced by 51.46% (10 min), 71.38% (30 min), 82.46% (60 min) and 85.81% (120 min) on average, across all pH groups. The reduction in CTC is similar to the findings of Sorour et al. (24), who reported a 91.9% reduction in CTC after wet-heating a high tannin sorghum grain for 2 h. Other studies using wet-heating also saw a 54% reduction in CTC after 10 min of heating (23). Additional high-moisture heat methods were examined during tea processing, where the CTC of two sorghum grains decreased by 22 and 56% (26). The effect of pH and heat treatments on the condensed tannic content in sorghum is not commonly reported in literature.

#### 4.2.3 HPLC analysis

As previously mentioned, there is limited literature investigating wet-heating and pH effects on sorghum phenolics. Therefore, comparisons to studies examining other plant derived phenolics have been evaluated for the purpose of this discussion. Caffeic acid increased in concentration after 30 min of heating. Caffeic acid was the only cinnamic acid to consistently increase with heating. Ferulic acid only increased in content after 60 min of heating for pH 8. The increase in both phenolic compounds from wet-heating is unexpected but could be attributed to increased extractability (27). Hong et al. (28) reported decreases in caffeic and ferulic acids after wet-heating Quingke (barley) for 40 min. The last cinnamic acid, p-coumaric acid, decreased by 39% in unheated samples when compared to samples heated for 60 min at pH 3. This result was in line with Hong et al. (28), who reported a 26% loss in p-coumaric acid after wet-heating.

All three detectable 3-DAs significantly decreased after heating for 10 min, regardless of pH. This agrees with other research groups that used wet-heating methods on whole grain sorghum and saw significant decreases in apigenidin, luteolinidin, and 7-methoxy-apigeninidin. The authors hypothesized the decrease in 3-DA content was likely due to the 3-DAs leaching out during heating (29). In our study, however, whole flour is heated with pH buffers in a closed environment, and the whole sample was lyophilized. This prevents potential 3-DAs from leaching out during the heat process, and indicates the change in 3-DA content was likely caused by the thermal processing and not leaching out. Although initial apigenidin content decreased after 10 min, apigenidin content did not significantly decrease with heat time in lower pH levels (pH 3 and 5). While an initial decrease is observed, the lack of change in content with increased heat time suggests that some 3-DAs present in sorghum are more thermally stable at lower pH levels. This is in agreement with Yang et al. (30), who reported 3-DAs from sorghum maintained thermal stability over a range of pH levels. It was further stated by the authors that the type of acid or base used also plays an important role in determining 3-DA heat stability.

To date, studies on stability of flavones in sorghum during wet-heating are unavailable. Therefore, other heating methods were evaluated. The flavone, luteolin, was not detected in unheated samples, until the samples were heated for 30 min or more. This disagrees with the findings from Cardoso et al. (31), who saw a 53% decrease in luteolin after using a conventional oven. Differences in luteolin content may be due to differences in sorghum variety, as well as heating method and moisture content.

Another detected flavone, apigenin, also increased in concentration with heat time across all pH levels. Interestingly, apigenin was already detectable in raw sorghum samples prior to heating, and only significantly increased after 30 min of heating. This disagrees with Cardoso et al. (31), who reported a loss of 50% after using dry heat. The reported differences in luteolin and apigenin content may be explained by the use of different processing types (dry vs. wet-heating) and need to be examined more closely.

Catechin was detected in unheated samples and decreased in content after heating for 10 min. Interestingly, catechin did not continue to decrease with heating time. Rather, catechin increased in content from the 10 to 30 and 60 min time groups. After 30 min of heating, the catechin content was similar to the unheated samples. This trend is in alignment with other research groups who studied eight of the most common catechin standards found in teas under similar heating conditions (60–90°C). After heating solutions at 90°C for 8 h, it was reported that non-epistructured catechin compounds increased in concentration due to epimerization of other compounds into catechin, which was found to take place between 60 and 90°C (32). This process likely explains the increase in catechin content after the initial decrease.

Naringenin decreased in content after 10 and 60 min of heating at pH 5. However, both the higher and lower levels pH values remained unchanged in final concentration after 60 min of heating. Approximately 20 μg/g of naringenin was detected in sorghum, while 37 μg/g was reported in wet-heated barley (28). Naringenin, like eriodyctiol, indicates some heat stability, given the lack of significant change over time at specific pH levels. This is further supported by Chaaban et al. (33), who studied the heat stability of naringenin standards against various temperatures and times and saw little reduction in concentration.

Initial quercetin concentration of raw samples was not detectable. After wet-heating, however, quercetin content increased across all pH levels. The increase in quercetin content after heating may be attributed to the cleavage of quercetin from the cell walls or other macronutrients present during the heating process. Thermal processing can separate bound phenolics from plant cell walls or other macronutrients present in the food matrix (34). This may explain why quercetin was detected in the heated samples and not the unheated samples.

Taxifolin was the only detected flavononol and was not affected by heat or pH treatments. This is similar to other flavonoids in our study. In fact, with the exception of 3-DAs, all flavonoid groups either increased in content or were relatively unaffected by heating at various pH levels. Our findings suggest that sorghum flavonoids may be pH- and heat-stable in excess moisture environments; however, further research is needed to understand this relationship.

Literature examining the effects of thermal processing on specific sorghum phenolic compounds is limited and should therefore be studied further. It was previously reported that roasting red sorghum decreased phenolic content (35), which is a different process than cooking in excess moisture. Nonetheless, similar reductions were observed for the majority of the polyphenols studied. It is important to note that all changes in phenolic compounds reported in this study are from one sorghum genotype. Additionally, the phenolics were extracted in 70% (v/v) ethanol. While changes in phenolic content may be from the pH or high-moisture heat treatments, it may also be affected by the individual phenolic compound’s extractability in 70% ethanol.

### 4.3 Cell bioactivity

The effect of heating and high-moisture pH treatments on sorghum polyphenol content and its anticancer activity is not currently well reported. In our study, anticancer activity was more effective at lower pH levels. This is in line with the findings from Cox et al. (6), who studied the effects of different ethanol-based extraction conditions and their effect on human cancer cell inhibition. It was observed that solvents with lower pH values had greater cell inhibition rates (6). The increased cell inhibition at lower pH values may be explained by changes in plant phenolic structure and stability at different pH levels (25). Altering the structure of phenolic compounds may affect their anticancer activity. However, more research is needed to understand the effect of structural changes and their relationship to anticancer activities.

Pearson’s correlation factor of 1 are indicators of high positive correlations among trends, while a value of −1 indicates relationships that are strongly inversely related. The findings from our study suggest that TPC and CTC are both positively correlated with SW480 and HCT 116 cells. More studies with isolated condensed tannin and isolated non-condensed tannin polyphenols are needed to understand specific correlation among cell lines.

## 5 Conclusion

The objective of this study was to determine the effects of heating time and pH on high polyphenol sorghum for starch digestibility, phenolic profile and cell bioactivity in a high moisture environment. Heating consistently enhanced the starch digestibility of the sorghum samples, irrespective of pH treatment and time. Notably, total phenolic content and bioactivity exhibited minimal alterations following a 10 min heating duration. These results hold promising implications for the development of high-phenolic sorghum products and provide valuable insights to guide forthcoming animal and clinical studies. The demonstrated impact of wet-heating on starch digestibility, coupled with the preservation of phenolic content and bioactivity, underscores the potential of incorporating high-phenolic sorghum lines in future functional food formulations.

## Data availability statement

The raw data supporting the conclusions of this article will be made available by the authors, without undue reservation.

## Ethics statement

Ethical approval was not required for the studies on humans in accordance with the local legislation and institutional requirements because only commercially available established cell lines were used.

## Author contributions

JP: Conceptualization, Formal analysis, Investigation, Methodology, Visualization, Writing – original draft, Writing – review & editing. AS: Formal analysis, Investigation, Methodology, Visualization, Writing – original draft, Writing – review & editing. SC: Formal analysis, Investigation, Methodology, Visualization, Writing – original draft, Writing – review & editing. MP-F: Formal analysis, Investigation, Methodology, Writing – original draft, Writing – review & editing. JC: Formal analysis, Investigation, Methodology, Writing – original draft, Writing – review & editing. RP: Resources, Writing – original draft, Writing – review & editing. SB: Conceptualization, Formal analysis, Funding acquisition, Investigation, Methodology, Project administration, Supervision, Writing – original draft, Writing – review & editing. SW: Conceptualization, Formal analysis, Funding acquisition, Investigation, Methodology, Project administration, Supervision, Writing – original draft, Writing – review & editing. WW: Supervision, Writing – original draft, Writing – review & editing. DS: Conceptualization, Formal analysis, Funding acquisition, Investigation, Methodology, Project administration, Supervision, Writing – original draft, Writing – review & editing.
