# Supplementary figures and images for: Impact of heat and high-moisture pH treatments on starch digestibility, phenolic composition, and cell bioactivity in sorghum (Sorghum bicolor L. Moench) flour
Source: Front Nutr. 2024 Aug 8;11:1428542. doi: 10.3389/fnut.2024.1428542 (PMC11338920; doi:10.3389/fnut.2024.1428542)

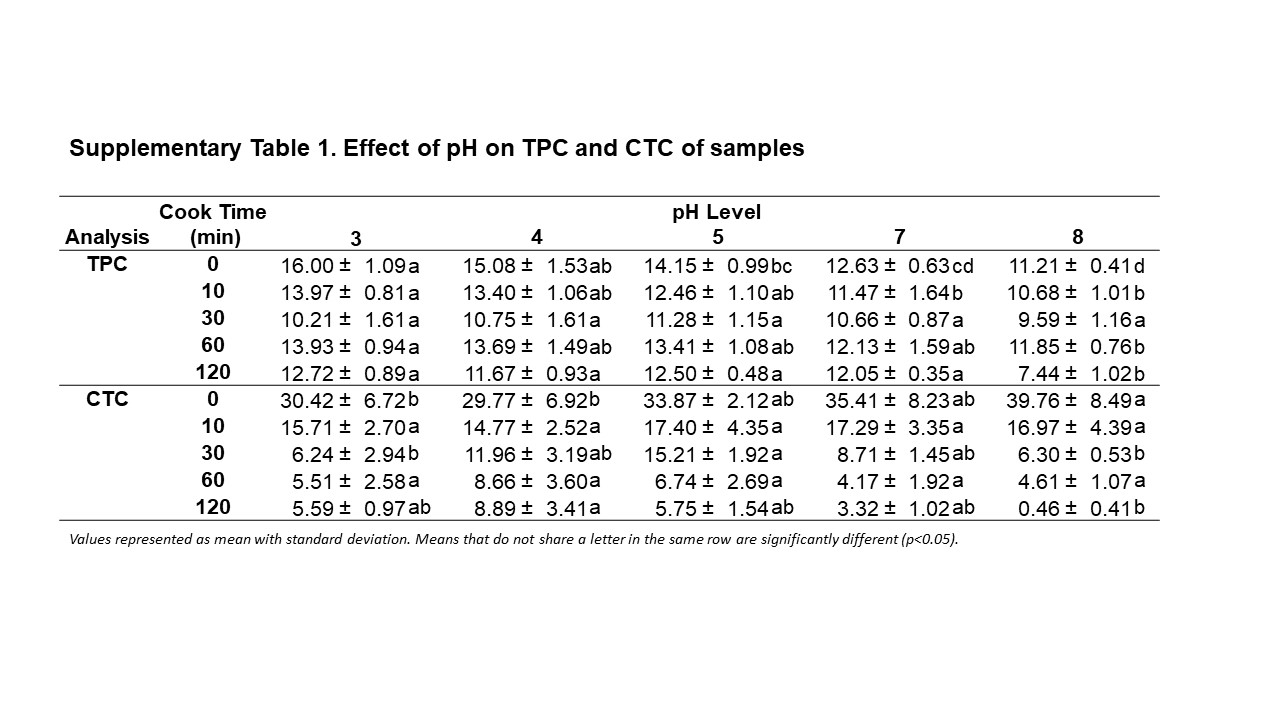

Supplement: Supplementary file 1 [file Image_1.JPEG]

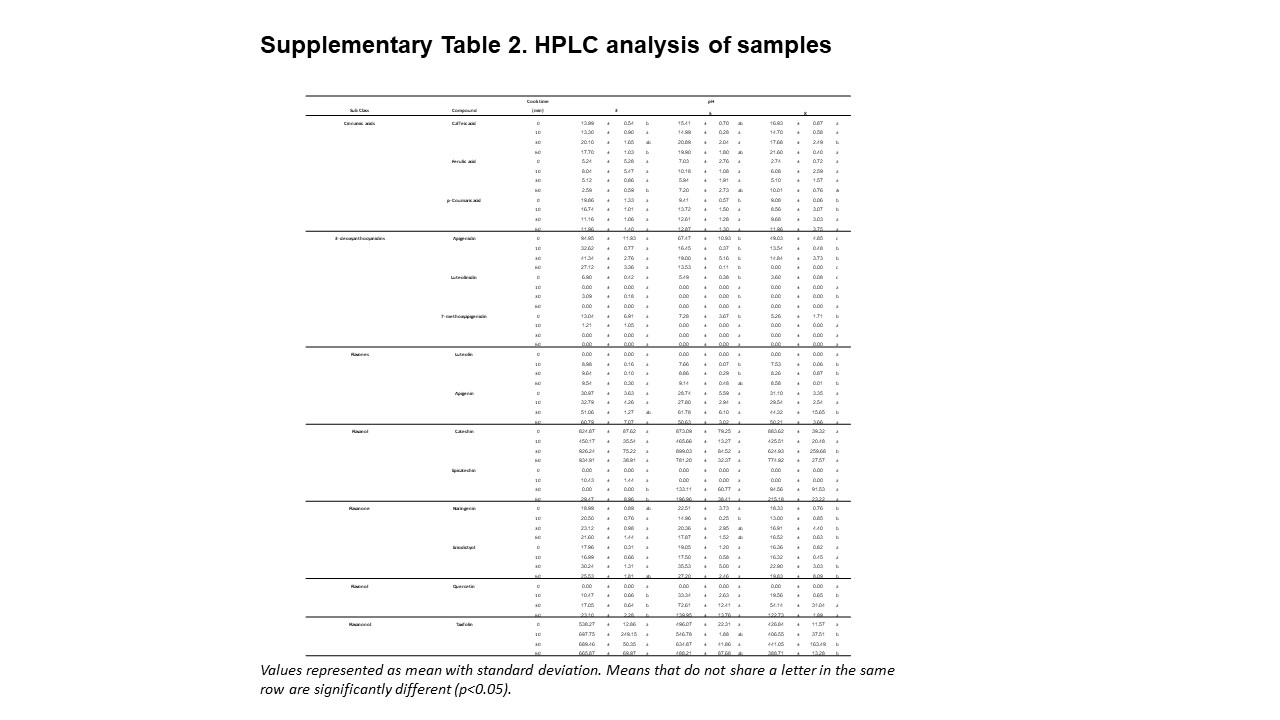

Supplement: Supplementary file 2 [file Image_2.JPEG]

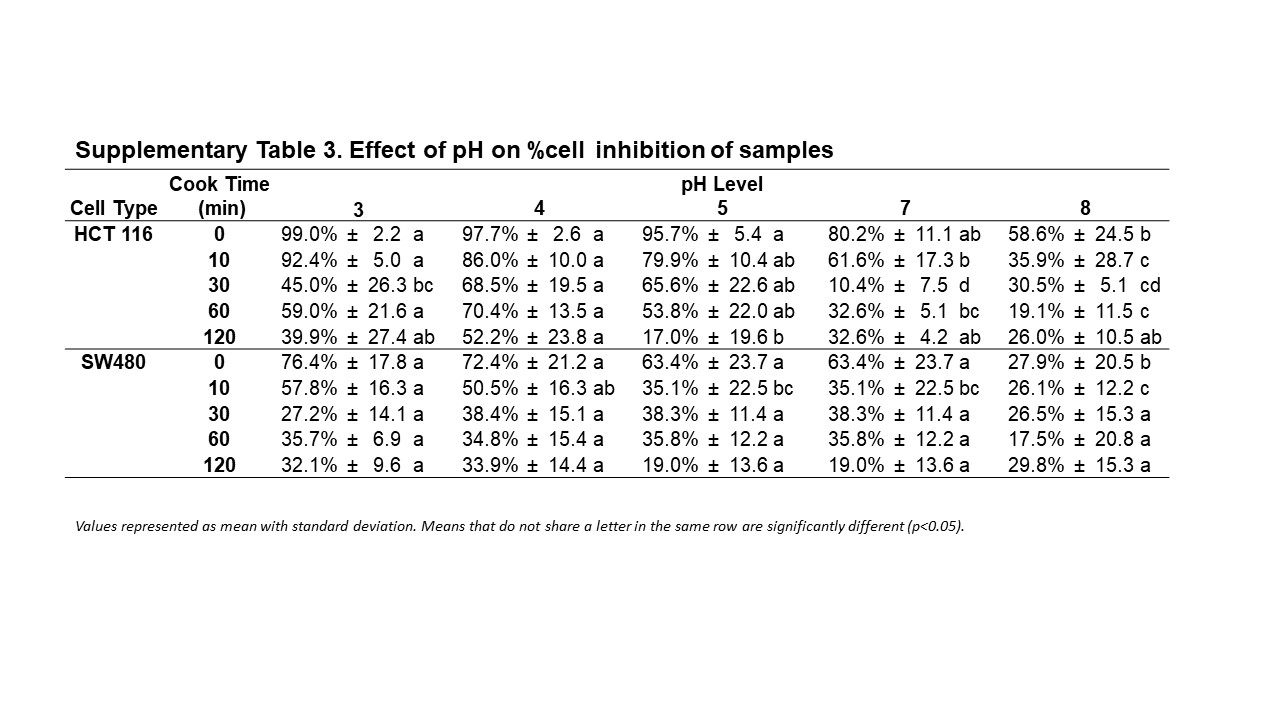

Supplement: Supplementary file 3 [file Image_3.JPEG]
